# Calcium Deregulation in Neurodegeneration and Neuroinflammation in Parkinson’s Disease: Role of Calcium-Storing Organelles and Sodium–Calcium Exchanger

**DOI:** 10.3390/cells13151301

**Published:** 2024-08-04

**Authors:** Guendalina Bastioli, Silvia Piccirillo, Laura Graciotti, Marianna Carone, Giorgia Sprega, Omayema Taoussi, Alessandra Preziuso, Pasqualina Castaldo

**Affiliations:** 1 Division of Neuroscience, San Raffaele Scientific Institute, 20132 Milan, Italy; bastioli.guendalina@hsr.it; 2 Department of Biomedical Sciences and Public Health, School of Medicine, University “Politecnica Delle Marche”, Via Tronto 10/A, 60126 Ancona, Italy; l.graciotti@staff.univpm.it (L.G.); marianna.carone@chem.ethz.ch (M.C.); s1112352@pm.univpm.it (G.S.); omayema.taoussi@gmail.com (O.T.); a.preziuso@staff.univpm.it (A.P.); 3 Institute of Chemical and Bioengineering, Department of Chemistry and Applied Biosciences, ETH Zurich, 8092 Zürich, Switzerland

**Keywords:** neurodegeneration, neuroinflammation, calcium dysregulation, sodium–calcium exchanger

## Abstract

Parkinson’s disease (PD) is a progressive neurodegenerative disorder that lacks effective treatment strategies to halt or delay its progression. The homeostasis of Ca^2+^ ions is crucial for ensuring optimal cellular functions and survival, especially for neuronal cells. In the context of PD, the systems regulating cellular Ca^2+^ are compromised, leading to Ca^2+^-dependent synaptic dysfunction, impaired neuronal plasticity, and ultimately, neuronal loss. Recent research efforts directed toward understanding the pathology of PD have yielded significant insights, particularly highlighting the close relationship between Ca^2+^ dysregulation, neuroinflammation, and neurodegeneration. However, the precise mechanisms driving the selective loss of dopaminergic neurons in PD remain elusive. The disruption of Ca^2+^ homeostasis is a key factor, engaging various neurodegenerative and neuroinflammatory pathways and affecting intracellular organelles that store Ca^2+^. Specifically, impaired functioning of mitochondria, lysosomes, and the endoplasmic reticulum (ER) in Ca^2+^ metabolism is believed to contribute to the disease’s pathophysiology. The Na+-Ca^2+^ exchanger (NCX) is considered an important key regulator of Ca^2+^ homeostasis in various cell types, including neurons, astrocytes, and microglia. Alterations in NCX activity are associated with neurodegenerative processes in different models of PD. In this review, we will explore the role of Ca^2+^ dysregulation and neuroinflammation as primary drivers of PD-related neurodegeneration, with an emphasis on the pivotal role of NCX in the pathology of PD. Consequently, NCXs and their interplay with intracellular organelles may emerge as potentially pivotal players in the mechanisms underlying PD neurodegeneration, providing a promising avenue for therapeutic intervention aimed at halting neurodegeneration.

## 1. Introduction

Neurodegeneration encompasses a broad spectrum of disorders affecting the neural system, each with distinctive etiologies and clinical manifestations. A common characteristic observed across these diseases, including PD, is the gradual and progressive loss of neurons and neuronal functions. Evidence has shown that neuroinflammation and the dysregulation of Ca^2+^ are closely related to neurodegenerative diseases, significantly contributing to the development and progression of neurodegeneration [1]. PD is an extremely heterogeneous disorder; approximately 1% of the population will develop the disease by age 65 and the prevalence rises to nearly 5% by age 85. PD is the second most common neurodegenerative disorder after Alzheimer’s disease [2]. The neurodegeneration observed in PD usually progresses slowly and chronically, with an average duration of about 15 years from the time of diagnosis to death. However, many individuals with PD can live for more than 20 years after being diagnosed [3]. The neuropathological features of PD include the progressive degeneration of dopaminergic neurons in the substantia nigra pars compacta (SNpc) and the buildup of intracellular inclusions within neurons and glial cells, predominantly made up of the presynaptic protein α-synuclein (α-syn). The striatum, which consists of the caudate and putamen nuclei, primarily receives projections from the dopaminergic neurons of the SNpc. Consequently, the accumulation of α-syn and the death of nigral cells reduce striatal dopamine (DA) levels. Neuroinflammation significantly contributes to the series of events that result in cell death in PD [4]. In this context, it has been noted that PD brains exhibit a significant glial response and signs of neuroinflammation, characterized by the activation of microglial cells and T lymphocytes. The decrease in nigrostriatal input increases the inhibitory output from the globus pallidus interna to the thalamus and, indirectly, to the cortex, thereby suppressing the initiation of movements. The disease is marked by both motor symptoms, such as bradykinesia, rigidity, resting tremor, and postural instability, as well as non-motor symptoms, including depression, anxiety, fatigue, sleep disturbances, and cognitive disorders [2,3]. Current evidence indicates that 15% of PD patients have a family history of the condition, and 5–10% exhibit a monogenic form of the disease with Mendelian inheritance. Nevertheless, the majority of PD cases are sporadic and have an unknown cause, likely involving a mix of genetic and environmental factors [5,6]. The HUGO Gene Nomenclature Committee (HGNC) identified and named at least 23 loci and 19 genes associated with parkinsonism. Although many forms of environmental exposure have been studied for their association with PD risk, the epidemiological connections with these factors are not fully understood. However, it is well established that individuals with occupational exposure to chemicals such as pesticides have a higher risk of developing PD [7]. The hypothesis that exposure to pesticides and other environmental chemicals is linked to an increased risk of PD arose from studies on the neurotoxic effects of a metabolite of 1-methyl-4-phenyl-1,2,3,6-tetrahydropyridine (MPTP) [3,8]. In particular, a connection has been established between the risk of developing PD and exposure to pesticides that affect mitochondrial complex I (such as rotenone) or induce oxidative stress (such as paraquat). While the neurotoxic effects of heavy metals like manganese and iron have been documented, their association with an increased risk of PD remains controversial [3,8,9,10]. Other studies have shown that potential risk factors relating to PD also encompass traumatic brain injury, cigarette smoking, ß-blocker use, and caffeine [3,11,12,13,14,15,16]. However, Ross and coauthors found that high coffee and caffeine intake was associated with a significantly lower incidence of PD [17]. To date, Levodopa (L-dopa) is considered “the gold standard” treatment for motor symptoms; however, chronic use is often associated with several motor complications. Other agents targeting dopaminergic pathways (i.e., dopaminergic agonists, monoamine oxidase B (MAO-B), selegiline, and rasagiline) can positively affect dopaminergic neurotransmission, thus ameliorating PD-related symptoms [18].

At the cellular level, multiple neurodegenerative processes have been identified in the death of dopaminergic neurons in PD. These include disrupted proteostasis, Ca^2+^ signaling abnormalities, mitochondrial dysfunction, impaired vesicle trafficking, lysosomal dysfunction [19,20], endoplasmic reticulum (ER) stress, and abnormal protein degradation by the ubiquitin–proteasome system [21]. Moreover, the activation of neuroinflammation response results in the release of pro-inflammatory mediators, including increased cytokine levels and the upregulation of inflammatory-associated factors like cyclooxygenase-2 and inducible nitric oxide synthase [22].

Recently, mounting evidence has increasingly pointed towards the involvement of Ca^2+^ dysregulation in the pathogenesis of PD [23,24]. This dysregulation involves different mechanisms of neurodegeneration and neuroinflammation; in particular, one of the most important regulators of Ca^2+^ homeostasis is the Na^+^-Ca^2+^ exchanger (NCX), which is an antiporter membrane protein that removes Ca^2+^ from cells.

Given the importance of Ca^2+^ homeostasis in neurodegenerative diseases, particularly in PD, this review examines the literature regarding the role of NCX in DA neurons and glia cells, as well as mitochondrial NCX, within PD models.

## 2. Ca^2+^ Deregulation in PD

Ca^2+^ serves as a crucial second messenger in all eukaryotic cells, orchestrating essential cellular activities. It is especially crucial in neurons, where it is involved in transmitting depolarizing signals and enhancing synaptic plasticity—an integral process for learning and memory—as well as the regulation of specific gene expressions. Two major forms of synaptic plasticity, long-term potentiation (LTP) and long-term depression (LTD), are cellular mechanisms involved in learning and memory [25]. These processes are triggered by the coupling between plasma membrane depolarization and the increase in intracellular Ca^2+^ [Ca^2+^]i. [25]. This elevation in [Ca^2+^]i involves various mechanisms, including the release of Ca^2+^ from Ca^2+^-storing organelles, such as mitochondria, ER, and lysosomes [26,27,28]. A diverse pattern of specialized Ca^2+^ pumps, channels, and Ca^2+^-binding proteins are involved in the regulation of cellular Ca^2+^ signaling, ensuring the maintenance of cellular homeostasis and the performance of specific cellular functions [26,27,28]. Ca^2+^ is also involved in the regulation of metabolism through enzyme phosphorylation and dephosphorylation and motility processes, such as those related to the cytoskeleton, the secretion of molecules via exocytosis, the transcription of numerous genes, and the process of programmed cell death [29]. The proper functioning of all these processes is essential for neuronal viability. However, during the processes of neurodegenerative disease, the capacity of neurons to maintain adequate energy levels can be compromised, thus impacting overall Ca^2+^ homeostasis and Ca^2+^-related signaling functions [29].

Two organelles predominantly involved in Ca^2+^ buffering are the ER and mitochondria; in addition, in recent years, it has been demonstrated that interactions between lysosomes and ER compartments could generate relevant global Ca^2+^ signals within the cell [28,30]. To date, it has been shown that unbalanced mitochondrial Ca^2+^ homeostasis is pathogenically linked to the neurodegeneration that occurs in PD [31,32]. Recent studies indicate that elevated mitochondrial Ca^2+^ can trigger the opening of the permeability transition pore and initiate apoptosis [33]. Interestingly, Ca^2+^ directly promotes α-syn aggregation [34,35], thereby increasing its toxicity, and it also impairs lysosomal motility and the turnover of misfolded proteins [36,37,38].

Neurons, as excitable cells, typically maintain a membrane potential of around −70 mV (varying by neuronal type), which is regulated by the exchange of specific ions, such as Na^+^, K^+^, Ca^2+^, and Cl^−^, between extracellular and intracellular environments. Neurons respond to small changes in input currents by generating action potentials at various frequencies [29,39,40,41,42]. The diversity in neuronal responses arises from the differential expression of distinct voltage-dependent channels responsible for conducting Na^+^ and voltage-dependent Ca^2+^ currents, as well as voltage-gated K^+^ currents and Ca^2+^-activated K^+^ currents. These channels exhibit varying selectivity and sensitivity, which is influenced by their specific subunit composition [43].

Nevertheless, neurons have traditionally been considered the sole excitable cells of the nervous system due to their ability to generate action potentials that can be transmitted to other neurons through synaptic transmission. Recent studies have clearly established that the generation of specific rapid signals, which are transmitted to other cells, is not an exclusive characteristic of neurons. Astrocytes, while not capable of generating action potentials, exhibit fast, transient changes in their ionic content. Moreover, these ionic signals can spread to neighboring cells, either passively or actively [44]. Extensive fluctuations in Ca^2+^ levels within neurons can lead to the overstimulation of various ionic channels and transporters. These include L-type voltage-gated Ca^2+^ channels (Cav1 family), specifically Cav1.2 and Cav1.3. While Cav1.2 is predominantly found in juvenile SNpc, DA neurons, Cav1.3 becomes more prominent in aging SNpc DA neurons in terms of Ca^2+^ influx and supporting rhythmic pacemaking activity [23,29,43,45,46,47]. Additionally, cyclic nucleotide-sensitive channels, as well as plasma membrane transporters, such as Na^+^-Ca^2+^ exchangers (NCXs) and mitochondrial Na^+^-Ca^2+^ exchangers (mitoNCXs), are also involved [48,49,50].

## 3. Neurodegeneration and NCX: Interplay in PD

The SLC8 gene family, which encodes NCX, includes several proteins [51,52,53]. In mammals, three different SLC8 genes have been identified: SLC8A1, which encodes NCX1 protein [54]; SLC8A2, which encodes NCX2 [55]; and SLC8A3, which encodes NCX3 [56,57]. The three isoforms of NCX1-3 are expressed in a tissue-specific manner [51,52] across both excitable and non-excitable tissues [58,59,60]. NCX1 and its various splicing isoforms have been extensively studied and are found in nearly every mammalian cell [51,55,61,62,63]. NCX2 and NCX3 have been identified in the brain and skeletal muscle, with NCX2 being cloned from rat brains in 1994 and NCX3 being cloned in 1996 [51,56,64].

NCX plays a crucial role in maintaining Ca^2+^ homeostasis within both plasma membrane and mitochondrial compartments by facilitating the bidirectional flow of Na^+^ and Ca^2+^. It operates in two modes: forward mode (Ca^2+^ efflux/Na^+^ influx) and reverse mode (Ca^2+^ influx/Na^+^ efflux) [60,65]. It has a stoichiometry of three Na^+^ ions to one Ca^2+^ ion, creating an electrogenic current [66]. Due to the importance of intracellular Ca^2+^ balance and the significant role of NCX in Ca^2+^ regulation, the exchanger has been extensively studied in various pathological contexts [49,65,67,68,69,70,71,72,73,74,75]. Specifically, different NCX isoforms (NCX1, NCX2, NCX3) exhibit specific roles depending on the pathological conditions in which they operate [67,76,77].

The distribution of NCX1, NCX2, and NCX3 in the CNS of rats has been extensively examined by various research groups (Table 1) [65,78]. Studies have shown that NCX1 transcripts are highly expressed in the motor area of the cerebral cortex, particularly in the pyramidal neurons of layers III and V, as well as in the thalamus, CA3 and dentate gyrus regions of the hippocampus, certain hypothalamic nuclei, and the cerebellum [65]. NCX2 transcripts are predominantly found throughout all hippocampal subregions, the striatum, and the paraventricular thalamic nucleus. NCX3 transcripts are mainly detected in the hippocampus, thalamus, amygdala, and cerebellum.

Regarding protein expression, immunohistochemical analysis has shown that NCX1 is present in the striatum (the terminal projection field of dopaminergic nigrostriatal neurons), the supragranular layers of the cerebral cortex, the hippocampus, the hypothalamus, the SNc and the ventral tegmental area, and the granular layer of the cerebellum. NCX2 is primarily located in neurons of the somatosensory cortical area, the neurons of layers V and VI of the sensory cortex, and the hippocampus, striatum, thalamus, and hypothalamus. NCX3 protein expression is especially prominent in the CA3 subregion, the oriens, radiatum, lacunosum–moleculare layers of the hippocampus, the nucleus accumbens (a brain area involved in the motivational control of motor coordination), and the molecular layer of the cerebellum [65].

Growing evidence suggests that the disruption of Ca^2+^ homeostasis, at both cytosolic and mitochondrial levels, might contribute to the pathophysiology of neurodegenerative diseases, including PD, and NCX has recently gained much attention.

### NCX in Neurons

In a PD model induced by MPTP, which causes dopaminergic loss and behavioral impairment similar to PD symptoms [79], the involvement of NCX was examined. The specific NCX inhibitor SEA0400 (Taisho Pharmaceutical Co., Ltd., Saitama, Japan) was found to improve MPTP-induced motor coordination impairment and attenuate both reduced DA levels and tyrosine hydroxylase (TH) immunoreactivity in the SNpc and striatum [80]. In a recent study involving transgenic mice bearing the α-syn A53T human mutation, it was observed that the three NCX1-3 isoforms were modulated differently. Specifically, the protein levels of NCX3 were reduced in the midbrain of A53T mice, whereas the level of NCX1 and NCX2 remained unaltered as compared to wild-type (WT) mice. However, in the striatum, this study showed increased NCX1 protein levels [81]. In particular, it is interesting to point out that the reduction in NCX3 expression in dopaminergic neurons in the SNpc of A53T mice was associated with elevated cytosolic and mitochondrial Ca^2+^ concentrations and increased neuronal death. These results support the hypothesis that alterations in the expression and activity of NCX1 and NCX3 proteins in different regions of the dopaminergic nigrostriatal circuit may disrupt intracellular Ca^2+^ levels, contributing to neuronal loss in PD [23,81].

In our recent work, we found that in both ex vivo and in vitro models of PD, co-exposure to α-syn and rotenone resulted in Ca^2+^ dysregulation and disruption in synaptic transmission and cell function. Notably, while the selective pharmacological inhibitor SN-6, which targets plasma membrane NCX1, did not prevent changes in striatal electrical activity or Ca^2+^ dysregulation, the inhibition of mitochondrial NCX using CGP-37157 completely reversed the neurotoxic effects of α-syn and rotenone exposure [71]. This topic will be examined in more detail in “NCX in Mitochondria in PD”.

In the same in vitro PD-mimicking model, it was observed that co-exposure to α-syn and rotenone has a detrimental impact on energy metabolism [72]. The provision of an alternative metabolic substrate like glutamate exerts a protective role by enhancing cell viability, increasing ATP levels, mitigating oxidative damage, and reducing Ca^2+^ overload [72]. Notably, the beneficial effects of glutamate were completely lost when the expression of either Excitatory Amino Acid Transport 3 (EAAT3) or NCX1 was silenced, highlighting the functional correlation between EAAT3 and NCX1 and the potential of targeting EAAT3/NCX1 function to mitigate PD pathology, as it facilitates glutamate uptake and metabolic utilization in dopaminergic neurons [72].

This evidence suggests a tight connection between the disruption of Ca^2+^ homeostasis and the development of neurodegenerative diseases such as PD. The existing literature indicates that prolonged intracellular Ca^2+^ elevation in brain cells could be a key early event in PD. While many underlying mechanisms remain to be elucidated, targeting NCX activity could offer a promising new strategy for preventing neuronal degeneration and cell death.

## 4. Dysregulation of Ca^2+^-Storing Organelles in PD (Mitochondria, ER, and Lysosomes)

**Figure 1:**
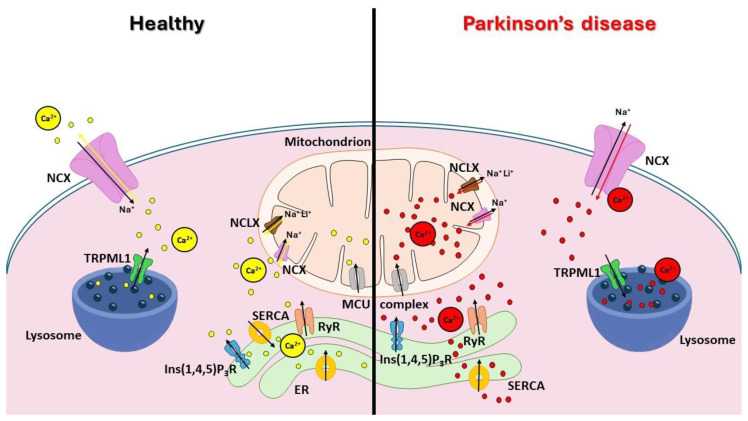
Regulation of Ca^2+^ homeostasis in physiological state and Parkinson’s disease (PD). In the physiological state (left panel), Ca^2+^ homeostasis is maintained through the balanced activity of various Ca^2+^ channels and exchangers. The endoplasmic reticulum (ER) functions as the primary intracellular reservoir for Ca^2+^ ions actively managed by sarco/endoplasmic reticulum Ca^2+^ ATPases (SERCAs) that pump Ca^2+^ into the ER. The controlled release of Ca^2+^ from the ER is mediated by ryanodine receptors (RyRs) and inositol 1,4,5-triphosphate receptors (Ins(1,4,5)P3Rs). Mitochondria, located in close proximity to the ER, capture the released Ca^2+^ through the mitochondrial Ca^2+^ uniporter (MCU) complex, thereby regulating cellular metabolism. Lysosomes are recognized as the second-largest reservoir of intracellular Ca^2+^. The lysosomal release of Ca^2+^ is mediated by TRPC mucolipin 1 (TRPML1), whose activity is critical for maintaining proper lysosomal membrane trafficking. The sodium–calcium exchanger (NCX) in both the plasma membrane and mitochondria functions normally, extruding Ca^2+^ in the forward mode and maintaining cellular Ca^2+^ balance; the sodium–calcium–lithium exchanger (NCLX) in the mitochondria operates effectively, contributing to the regulation of mitochondrial Ca^2+^ levels. Red arrows are related to Ca²⁺ influx, while black arrows indicate Ca²⁺ efflux.

As mentioned previously, evidence indicates that elevated Ca^2+^ influx at the plasma membrane level plays a significant role in PD pathogenesis, but Ca^2+^ deregulations at different intracellular levels are also implicated, with particular reference to the dysregulation of Ca^2+^ from intracellular Ca^2+^ stores [26,27,28]. The major intracellular Ca^2+^ stores are the ER and the mitochondria, and in recent years, important evidence has shown that other organelles, such as the lysosomes, also act as relevant intracellular Ca^2+^ reservoirs (Figure 1) [30].

### 4.1. Ca^2+^ Storage in Mitochondria

Mitochondria are intracellular organelles involved in various metabolic pathways, including the oxidation of carbohydrates and fatty acids, the Krebs cycle, and oxidative phosphorylation. They serve as the primary source of ATP production within cells. The intricate role of Ca^2+^ in regulating the bioenergetic functions facilitated by mitochondria has received extensive research [82]. The mitochondrial respiratory chain where the oxidative phosphorylation occurs is located within the inner membrane. The transport of electrons through the electron transport chain (ETC) uses the energy released from the transport of electrons to pump protons from the mitochondrial matrix into the intermembrane space (IMS) through complex I, III, and IV. This process creates a proton gradient and an electrochemical gradient across the mitochondrial inner membrane, resulting in a negative charge inside the membrane [83]. Mitochondrial Ca^2+^ uptake plays a crucial role in regulating cytosolic Ca^2+^ homeostasis, thereby modulating the activity of Ca^2+^ channels. Moreover, the mitochondria are one of the major cellular producers of reactive oxygen species (ROS). In the ETC, electrons occasionally captured by oxygen produce superoxide anion radicals (O^2−^), with complexes I and III being the main sites of superoxide production [84].

In neurons, Ca^2+^ transients play an important role in the generation of an action potential, releasing neurotransmitters and facilitating intracellular signaling [25,68,85]. Under normal conditions, the increase in intracellular Ca^2+^ concentrations is transient. The influx of extracellular Ca^2+^ through ion channels and transporters, or the release of Ca^2+^ from intracellular stores, elevates Ca^2+^ transients, which plays a significant role in neuronal activity [25,68,85]. Mitochondria located adjacent to the Ca^2+^ channels of the ER and plasma membrane protect against excess cytosolic Ca^2+^ by rapidly sequestering Ca^2+^ and modulating specific neuronal functions [25,68,85]. Mitochondria also play a crucial role in preventing disturbances in Ca^2+^ homeostasis through their buffering activity. Ca^2+^ accumulation in the mitochondrial matrix is mainly regulated by the mitochondrial Ca^2+^ uniporter (MCU) at the inner mitochondrial membrane (IMM). This process is driven thermodynamically by the negative membrane potential (Δψm) established by the respiratory chain. This accumulation is balanced by mechanisms that facilitate Ca^2+^ extrusion, including a Na^+^-dependent exchange system that controls Ca^2+^ efflux from the mitochondria. However, persistent intracellular Ca^2+^ concentrations can trigger downstream neurotoxic effects and stress conditions, such as those seen under different pathological conditions or in the case of aging. This can result in the uncoupling of mitochondrial electron transfer from ATP synthesis and the excessive activation of enzymes, protein kinases, and nitric oxide synthase, leading to increased oxidative stress and apoptosis [21,85,86,87].

Different studies have reported that specific mutations in genes, such as α-syn (SNCA), leucine-rich repeat kinase 2 (LRRK2), PARKIN, DJ-1, ATP13A2, and PTEN-induced kinase 1 (PINK1)—along with exposure to environmental toxins—can inhibit complex I of the mitochondrial respiratory chain. This inhibition results in the overproduction of ROS, which is considered a significant risk factor for the development of idiopathic PD [3,31,88]. Previous studies observed that oligomeric structures of α-syn can dramatically affect mitochondrial Ca^2+^ homeostasis, compromising neuronal function. In particular, α-syn oligomers can interact with both ATP synthase and complex I, causing mitochondrial Ca^2+^ overload, oxidative stress, and the disruption of organelles, including mitochondria, ER, and lysosomes [89,90,91,92,93]. Mutations that alter the function of Parkin or PINK1, pivotal regulators of mitochondrial balance, lead to the accumulation of damaged mitochondria. This accumulation intensifies oxidative stress and triggers the release of damage-associated molecular patterns (DAMPs) [94]. A recent study reported that Ca^2+^ homeostasis disruptions could potentially lead to synaptic dysfunction by depleting Ca^2+^ levels and impairing energy production due to mitochondrial alterations and bioenergetic impairments [95]. They observed early-stage physiological disruptions in DA neurons derived from induced pluripotent stem cells (iPSCs) of patients carrying a mutation in the β-glucocerebrosidase (GCase) enzyme encoded by the GBA gene (GBA-N370S mutation), a prominent genetic risk factor for PD. These neurons exhibit persistent Ca^2+^ dysregulation, primarily in the mitochondria, along with subsequent reductions in mitochondrial membrane potential and oxygen consumption rate, all suggestive of mitochondrial dysfunction [95].

#### NCX in Mitochondria in PD

The mitochondrial Na^+^/Ca^2+^ exchanger (NCXmito) is the primary mechanism for Ca^2+^ extrusion in neurons and other excitable cells [68,96]. In 1974, Carafoli and colleagues discovered that incubating isolated heart mitochondria with 20–50 mM Na^+^ led to Ca^2+^ release, an effect specific to Ca^2+^ that was not observed with K^+^, Rb^+^, Cs^+^, or Mg^2+^ [97]. Subsequent investigations carried out by Crompton and his group in 1976 demonstrated that Ca^2+^ efflux occurred when mitochondria were exposed to high Na^+^ concentrations in the presence of ruthenium red, which inhibits the mitochondrial Ca^2+^ uniporter. They also found that Na^+^ can be replaced by Li^+^ to promote Ca^2+^ efflux [98]. Later, NCLX (Na^+^/Ca^2+^/Li^+^ exchange), a novel Na^+^/Ca^2+^ exchanger, was identified and characterized [52,99,100]. NCLX facilitates Na^+^- or Li^+^-dependent Ca^2+^ transport at similar rates, a characteristic shared exclusively with the unidentified mitochondrial exchanger [52,99,100]. Of note, evidence reported that an endogenous pool of plasma membrane NCX (NCX1-3) is also expressed within the mitochondria in various cell types [71,101,102,103,104,105,106]. Different studies investigating the cellular and subcellular distribution of NCX isoforms in the CNS of rats demonstrated that NCX-positive mitochondria are located within neurons and astrocytes [96,101,102]. Specifically, in astrocytes, NCX-positive mitochondria are mainly located in thick proximal processes, whereas in neurons, these NCX-positive mitochondria are predominantly found in dendrites and the neuronal soma rather than at the synaptic terminals [96,101]. NCX1-positive mitochondria were mainly observed in distal dendrites, while both NCX2- and NCX3-positive mitochondria were consistently distributed across all neuronal compartments [96,101,102]. In PD, the role of NCXmito remains largely unclear; however, several studies suggest that it could be a promising target for investigation. In this regard, Visch and coauthors used human complex I-deficient fibroblast cell lines derived from skin biopsies of 0–5-year-old patients with Leigh syndrome as a model for the oxidative phosphorylation system disease. This model showed a reduction in mitochondrial Ca^2+^ accumulation and decreased intracellular ATP levels. Using the inhibitor of NCXmito, CGP37157, they found increased mitochondrial Ca^2+^ accumulation and restored ATP synthesis [50]. Although these data are derived from children aged 0 to 5 years rather than PD patients, they suggest that CGP-37157 could be a promising approach for addressing mitochondrial dysfunction in PD [50]. In agreement with this, our study showed that exposure to α-syn plus rotenone on cortisol striatal slices of WT mice and SH-SY5Y cells promotes mitochondrial Ca^2+^ accumulation, which is prevented by either CGP-37157 or the silencing of NCX1 expression, thereby limiting mitochondrial Ca^2+^ uptake [71]. Other studies based on genetic mutations of PD have shown interesting evidence to support the crucial role of mitochondrial NCX and NCLX. It was demonstrated that, under physiological conditions, PINK1 controls Ca^2+^ efflux from the mitochondria through the mitochondrial NCX, while PINK1 deficiency leads to mitochondrial Ca^2+^ accumulation, ROS overproduction, and impaired respiration [107,108]. Consistent with these studies, it has been observed that in a PD model based on PINK1 knockout, the activity of NCLX is strongly compromised, leading to reduced mitochondrial Ca^2+^ efflux [109]. Interestingly, the activation of the protein kinase A (PKA) pathway fully restores NCLX function by phosphorylating serine 258, a potential regulatory site on NCLX. Of note, a constantly active phosphomimetic variant of NCLX prevents mitochondrial Ca^2+^ overload and depolarization in PINK1-deficient neurons, thereby promoting neuronal survival [109]. Accordingly, in another PD model based on LRKK2 deficiency, it was found that the deletion, inhibition, and mutations of LRRK2 impaired mitochondrial Ca^2+^ extrusion through NCLX, which, in turn, reduces the threshold for mitochondrial permeability transition pore opening and increases cell death. This effect can be mitigated by upregulating NCLX through either a direct or indirect cAMP/PKA-dependent mechanism [110]. These findings underscore the essential role of both NCX and NCLX, operating on the mitochondrial membrane, in regulating Ca^2+^ ion’s homeostasis in PD by extruding Ca^2+^ from mitochondria. Therefore, they represent promising therapeutic targets and warrant further investigation.

### 4.2. Ca^2+^ Storage in the ER

The ER performs several crucial roles, including protein synthesis, ensuring protein quality control, maintaining Ca^2+^ balance, and participating in lipid and carbohydrate metabolism [111,112].

The ER network is extensively interconnected with many organelles, including mitochondria, lysosomes, and other organelles, through Ca^2+^-dependent pathways [21,87,113,114,115,116,117,118,119,120,121]. Three types of proteins are highly expressed in the ER and play distinct roles in controlling Ca^2+^ homeostasis: IP3R and RyR are Ca^2+^ release channels that transport Ca^2+^ from the ER lumen to the cytoplasm, contributing to cellular Ca^2+^ signaling; ER-resident Ca^2+^-binding proteins determine the ER’s Ca^2+^ buffering capacity; and Ca^2+^-ATPase from the Sarco(endo)plasmic Reticulum (SERCA) transports cytosolic Ca^2+^ into the ER lumen. SERCA isoforms participate in various Ca^2+^ signaling mechanisms, including excitation–contraction coupling, excitation–secretion coupling, gene transcription, and apoptosis due to elevated cytosolic Ca^2+^ levels. Both Ca^2+^ release channels and SERCA pumps are sensitive to changes in cytosolic and ER luminal Ca^2+^ levels, as well as ROS [122]. The homeostasis of the ER can be disrupted by various conditions, such as Ca^2+^ depletion from its lumen and oxidative stress. Increasing evidence suggests that alterations in the ER–mitochondrial network may significantly contribute to the development of PD [123,124,125]. Indeed, alterations in ER–mitochondria communication, combined with abnormal protein degradation, can contribute to the pathophysiology of PD [124,125,126].

Given the essential role of ER in cellular proteostasis—handling protein production, delivery, and degradation [127]—disruptions in ER–mitochondria interactions have harmful effects on dopaminergic neurons [128,129]. Indeed, changes in Ca^2+^ content within the ER can disrupt protein folding processes that rely on Ca^2+^ as a cofactor. This leads to the accumulation of unfolded proteins, causing ER stress and triggering an unfolded protein response (UPR). Disturbances in protein folding, such as α-syn in PD, the disruption of cytosolic Ca^2+^ homeostasis, issues in protein biosynthesis, alterations in Ca^2+^-mediated signaling pathways, and the impairment of other organelle functions highly dependent on ER interactions are major factors contributing to neurodegenerative diseases [127,130].

This is particularly evident in autosomal recessive or dominant familial PD involving proteins encoded by specific genes, including SNCA, PARKN, DJ-1, PINK1, ubiquitin carboxyl-terminal hydrolase-1 (UCHL-1), LRRK2, phospholipase A2G6 (PLA2G6), and ATP13A2 [131,132,133,134,135,136]. Interestingly, a structural and functional defect of the ER–mitochondria contact interface has been observed in iPSC-derived neurons from both patients with PARK2 mutations and PARK2 KO mice [126]. Given the role of Parkin in regulating communication between the ER and mitochondria, this could be particularly significant for dopaminergic neurons in the SNpc, which are known to be highly susceptible to disturbances in Ca^2+^ regulation.

### 4.3. Ca^2+^ Storage in the Lysosomes

Lysosomes are small acidic organelles within intracellular compartments that degrade proteins, lipids, nucleic acids, and pathogens [137]. Traditionally recognized for their catabolic functions, recent research has revealed that lysosomes also serve as important Ca^2+^ storage compartments. They play a significant role in regulating global intracellular Ca^2+^ homeostasis, which is crucial for both physiological and pathological conditions [137,138,139]. Moreover, lysosomes continuously communicate and exchange ions with the main intracellular Ca^2+^ stores, particularly the ER and mitochondria [28]. Defects in the management and release of lysosomal Ca^2+^ have been recognized as primary causes of lysosomal storage disorders (LSDs), which play a significant role in neurodegenerative diseases. These disorders typically stem from malfunctions in essential components of lysosomal Ca^2+^ regulation, leading to Ca^2+^ imbalance. In particular, research indicates a vital role in PD for genes such as GBA1, which encodes the lysosomal enzyme glucocerebrosidase, as well as LRRK2 and ATP13A2, both of which are associated with disturbances in lysosomal Ca^2+^ homeostasis [140,141,142].

Moreover, in the neurodegenerative processes associated with PD, lysosomal Ca^2+^ dysfunction impairs autophagy. The mucolipin TRP channel 1 (TRPML1), also known as mucolipin-1, is a cation-permeable channel on lysosomal membranes, and its activity is crucial for autophagy regulation [28,143]. This channel is extensively found in the brain and various other tissues, where it significantly contributes to lysosomal storage, signal transduction, membrane transport, and acidic balance [144]. Research has demonstrated that enhancing lysosomal Ca^2+^ activity can clear α-syn buildup and safeguard human dopaminergic neurons carrying a mutation in the ATP13A2 gene (also known as PARK9) [142]. PARK9 encodes a lysosomal-type 5 P-type ATPase crucial for maintaining cation homeostasis [145,146]. The dysfunction of PARK9 results in impaired lysosomal function [147,148]. Interestingly, elevated TRPML1 levels have been shown to clear damaged mitochondria and eliminate excess ROS, thereby reducing neuronal toxicity [149,150]. Transcription factor EB (TFEB) controls the formation and exocytosis of lysosomes by triggering the release of endolysosomal Ca^2+^ via TRPML1 channels [151,152]; it has also been observed to be involved in preventing cell death in PD models [28,153,154,155,156]. Notably, in rats expressing α-syn, the overexpression of TFEB has been observed to protect against neurodegeneration by reducing α-syn oligomers and preserving lysosomal function [153]. Furthermore, boosting β-glucocerebrosidase activity—the lysosomal hydroxylase encoded by the GBA1 gene, the mutated form of which causes Gaucher’s disease, a lysosomal storage disorder [157]—has been demonstrated to reduce α-syn accumulation and mitigate dysfunction in lysosomal, mitochondrial, and neuronal compartments [140,158]. Therefore, impaired autophagy can worsen disease symptoms, indicating that targeting autophagy might be a promising therapeutic strategy for preventing and treating neurodegeneration associated with PD.

In the PD state (right panel), the regulation of Ca^2+^ homeostasis is strongly compromised, characterized by altered activity of these channels and exchangers, leading to increased intracellular Ca^2+^ levels and contributing to neuronal dysfunction and degeneration. NCX in the plasma membrane and in mitochondria becomes less efficient, impairing Ca^2+^ export and leading to Ca^2+^ overload. NCLX and the MCU in the mitochondria are dysregulated, resulting in impaired mitochondrial Ca^2+^ uptake and release, which compromises mitochondrial function. The SERCA shows reduced activity, decreasing Ca^2+^ reuptake into the ER and leading to the depletion of ER Ca^2+^ stores. Ins(1,4,5)P3R and RyR in the ER exhibit abnormal activity, causing excessive Ca^2+^ release from the ER. TRPML1 in the lysosomes is dysregulated, affecting lysosomal Ca^2+^ release and trafficking. All of these dysfunctional components contribute to the disruption of Ca^2+^ homeostasis, exacerbating neuronal damage.

## 5. Neuroinflammation and NCX: Interplay in PD

Microglia, the resident immune cells of the CNS, are part of the primary innate immune cell population [159,160] and act as the first line of defense against various insults. Under normal conditions, microglial cells actively monitor their surrounding environment, extending their branching processes and changing their morphology in response to chemical signals [161]. This includes increasing the size of their soma and retracting their thin cytoplasmic processes [162]. Microglial processes can also establish direct contact with neuronal synapses [163], which plays a crucial role in the formation and disruption of synapses, in the synchronization of neuronal networks in both adult and developing brains [164,165,166], and in the removal of apoptotic cells. Additionally, microglia can release neurotrophic factors and hormones into the extracellular space [167].

Given the essential role of microglial cells in brain function, pathological changes in these cells are understandably linked to the accelerated progression of various neurological diseases, including PD [168]. While microglial activation generally protects the brain, persistent or chronic activation can lead to permanent brain injury [169] and is considered a common cause of tissue damage in PD [170]. In most neurodegenerative disorders, microglia have been observed to increase their proliferation rate, likely due to their ability to secrete a variety of cytokines and chemokines. Notably, various Toll-like receptors (TLRs 1-9) are constitutively expressed in rodent and human microglial cells [171,172]. These receptors detect pathogen-associated molecular patterns (PAMPs) or damage-associated molecular patterns (DAMPs) [173,174] and respond promptly to these insults. The activation of downstream TLR pathways leads to the production of pro-inflammatory cytokines (such as TNF-α, IL-1β, IL-6) or type I interferons (IFN-I), which induces the release of IFN-β and chemokines. Furthermore, chronically activated microglia release ROS and excitotoxins, depending on the properties of the microenvironment [175].

The majority of PD research has primarily focused on understanding the pathological processes occurring within neurons, often overlooking the crucial role of astrocyte interactions with neurons and other CNS cells in neurodegeneration. Consequently, approaches centered solely on neurons have not yielded successful neuroprotective treatments for PD. It has been observed that, in PD, microglia become activated in response to various stimuli, including the accumulation of misfolded proteins like α-syn and neuronal damage. This activation triggers a neuroinflammatory response characterized by the release of pro-inflammatory cytokines, chemokines, and ROS. The chronic activation of microglia in PD is thought to contribute to the progressive degeneration of dopaminergic neurons in the SNc [176,177]. However, this persistent activation triggers a complex cascade of events wherein activated microglia exhibit a dual role. On the one hand, they engage in phagocytosis, clearing damaged neurons and protein aggregates; on the other hand, they release harmful substances that exacerbate neuronal damage [178,179,180]. Moreover, the activated state of microglia can instigate the recruitment of peripheral immune cells, including T lymphocytes, into the brain, thereby intensifying the inflammatory response within the CNS. In a study conducted by Rogers et al., activated microglia surrounding damaged or dead pigmented DA neurons were observed in the SNpc of PD patients [181]. Furthermore, postmortem analysis of PD samples revealed elevated levels of inflammatory mediators in both the striatum and SNpc, underscoring the widespread inflammatory processes associated with the disease. However, the role of glia in PD extends far beyond inflammation. It has been recently demonstrated that astrocytes derived from PD patients exhibit several characteristic features of the disease [182,183,184]. These changes have significant consequences, including bioenergetic alterations, Ca^2+^ dysregulation, and heightened cytokine release in response to inflammatory triggers [184]. In 2012, Barkholt and colleagues revealed that in monkeys expressing the A53T variant of α-syn, the degeneration of dopaminergic neurons is linked to chronic microgliosis in the midbrain [185]. Considering the essential role of astrocytes in regulating blood–brain barrier permeability, the secretion of neurotrophic molecules, and synaptic transmission, it has been reported that α-syn can induce the activation of microglia, which, in turn, convert resting astrocytes into neurotoxic-reactive astrocytes. This conversion negatively affects the balance of cytokines, resulting in DA neuron loss [186,187]. In agreement with these results, in an in vitro PD model based on astrocytic-neuronal co-cultures, it was observed that astrocytes derived from iPSCs of patients with LRRK2 mutations co-cultured with healthy DA neurons showed α-syn accumulation along with a reduced number of DA neurons. These results suggest that astrocytes may influence α-syn pathology, playing a crucial role in the survival of dopaminergic neurons in PD [188]. In a study conducted by Lev and colleagues [189], DJ-1 knockout mice exhibited increased neuronal vulnerability to 6-hydroxydopamine toxicity. The researchers found that astrocytes deficient in DJ-1 had compromised antioxidant mechanisms, which impaired their ability to protect neurons from 6-hydroxydopamine-induced damage. This finding highlights an altered interaction between astrocytes and neurons, underscoring the crucial role of DJ-1 in maintaining neuronal viability through astrocytic support.

Dysfunctional mitochondria within microglia can promote a pro-inflammatory phenotype, thereby worsening neurodegeneration [190,191]. Supporting this notion, recent research has highlighted that astrogliopathy significantly influences the development and progression of PD [192,193]. Specifically, astrocytes derived from iPSCs obtained from PD patients carrying a mutation in the LRRK2 gene exhibit fragmented mitochondrial structures, diminished cell sizes, disrupted Ca^2+^ signaling, and metabolic dysfunction [192].

Considering the close morphological and functional interplay between microglia, astrocytes, and neurons, alterations in Ca^2+^ signals within astrocytic processes could disrupt neuronal function and potentially lead to the development of neurodegenerative processes. Interestingly, different studies pointed out that abnormal dopamine-mediated Ca^2+^ signals in astrocytic processes represent a potential mechanism by which EAAT2 downregulation occurs, leading to elevated extracellular glutamate levels and subsequent neurodegeneration [194,195,196,197,198,199].

The involvement of neuroinflammation in PD has led to the exploration of potential therapeutic targets, with NCX emerging as a significant candidate. Considering that this exchanger has been implicated in the regulation of neuroinflammation and its modulation affects neuronal viability, NCX has gained considerable attention as a potential target in terms of PD therapy. In MPTP-induced dopaminergic neurotoxicity in mice, the upregulation of NCX activity has been observed to be stimulated by nitric oxide production and microglial activation, resulting in intracellular Ca^2+^ elevation and ROS generation [80]. In agreement with this, the co-expression of NCX1 with IBA-1 positive cells has been observed in the striatum of A53T mice, supporting the hypothesis of a potential association between NCX and microgliosis in PD [81]. Moreover, early-stage mitochondrial dysfunction in mesencephalic neurons of A53T-α-syn mice contributes to neuronal degeneration and activates microglial cells in the striatum. This microglial activation leads to the release of pro-inflammatory factors in the striatum, resulting in further glial activation and the progressive impairment of dopaminergic neuronal plasticity during the late stages of the disease [48].

Altogether, these findings emphasize the intricate role of glia cells in PD and how the glia-neuronal interplay can affect the pathogenesis of PD. In particular, we highlighted the contribution of NCX in this context, suggesting that targeting microglial activation and associated inflammatory pathways could be a promising therapeutic approach for mitigating neuronal degeneration and neuroinflammation in PD.

## 6. Conclusions

**Figure 2:**
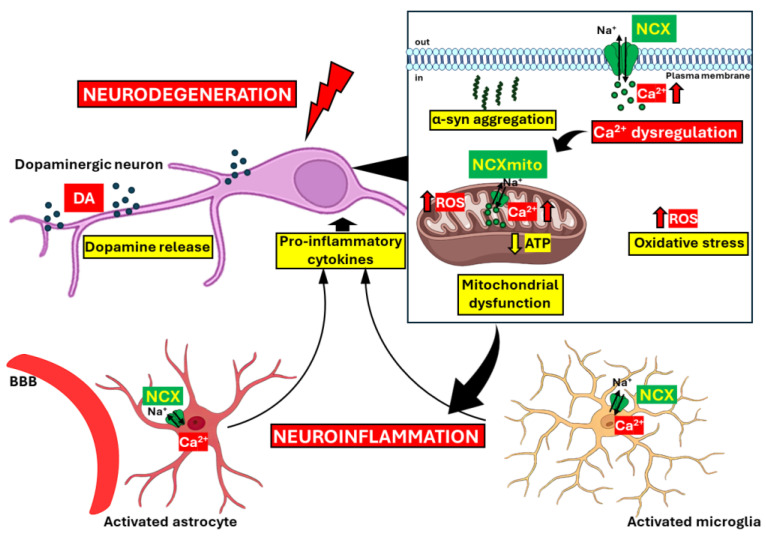
Schematic representation of the key neuropathological features of PD and the role of NCX. In PD, disrupted Ca^2+^ homeostasis, mitochondrial dysfunction, oxidative stress, misfolded protein aggregation, and neuroinflammation represent the key neuropathological features. NCX plays a crucial role as it becomes dysregulated and contributes to Ca^2+^ overload, exacerbating mitochondrial damage and neuronal cell death. Additionally, the accumulation of misfolded α-syn proteins and heightened oxidative stress further amplify both neurodegenerative and neuroinflammatory processes. DA = dopamine; BBB = blood–brain barrier; α-syn = α-synuclein; ROS = reactive oxygen species; NCX = Na^+^/Ca^2+^ exchanger.

PD is a multifactorial neurodegenerative disorder that affects millions of individuals worldwide, and, unfortunately, the exact mechanisms that regulate the onset of the disease are still unclear. Ca^2+^ signaling plays an important role in many aspects of neuronal function, and it is widely demonstrated that the dysregulation of Ca^2+^ homeostasis is a key feature of PD pathogenesis, strongly affecting neuronal viability and making it an important therapeutic target to study in terms of counteracting the progression of the pathology. The data reviewed here highlight the essential role of both plasma membrane and mitochondrial NCX in controlling Ca^2+^ homeostasis, underscoring the link between NCX, neurodegenerative processes, and neuroinflammation in PD. Due to the fact that neurons are highly vulnerable to Ca^2+^ perturbation and astrocytes play a regulatory role in neuronal and synaptic physiology, it is crucial to understand how Ca^2+^ signaling influences PD pathogenesis and the role of NCX in the progression of PD. Although several Ca^2+^ channels, transporters, and Ca^2+^-binding proteins are compromised in PD, the significant impact of NCX modulation in PD pathology suggests that regulating its activity may provide a promising avenue for in vivo experiments aimed at developing new therapeutic strategies. However, further detailed studies are necessary to elucidate the intricate relationship between the cross-talk of Ca^2+^ homeostasis, NCX, neuroinflammation, and Ca^2+^-storing organelles in PD (Figure 2).

## Figures and Tables

**Table 1:** Distribution of NCX isoforms in the brain.

NCX Isoforms		NCX Isoforms Expressed in Specific Brain Areas	NCX Isoforms Expressed in Specific Brain Cell Types	References
NCX1	mRNA	Neocortex (pyramidal neurons of the III and V layers), hippocampus (CA3 and the dentate gyrus), hypothalamic nuclei, cerebellum	Neurons, astrocytes, oligodendrocytes	[55,57,59,60,65,78]
	Protein	Striatum, cortex (supragranular layers), hippocampus, hypothalamus, substantia nigra, ventral tegmental area, cerebellum (granular layer)		
NCX2	mRNA	Hippocampus, striatum, paraventricular thalamic nucleus	Neurons, astrocytes, oligodendrocytes	
	Protein	Cortex (somatosensory area), hippocampus, striatum, thalamus, hypothalamus		
NCX3	mRNA	Hippocampus, thalamus, amygdala, cerebellum	Neurons, astrocytes, oligodendrocytes	
	Protein	Hippocampus (CA3 subregion, oriens, radiatum, lacunoso-moleculare layers), nucleus accumbens, cerebellum		

## References

[1] Guzman-Martinez L., Maccioni R.B., Andrade V., Navarrete L.P., Pastor M.G., Ramos-Escobar N. (2019). Neuroinflammation as a Common Feature of Neurodegenerative Disorders. Front. Pharmacol..

[2] Obeso J.A., Stamelou M., Goetz C.G., Poewe W., Lang A.E., Weintraub D., Burn D., Halliday G.M., Bezard E., Przedborski S. (2017). Past, present, and future of Parkinson’s disease: A special essay on the 200th Anniversary of the Shaking Palsy. Mov. Disord..

[3] Shulman J.M., De Jager P.L., Feany M.B. (2011). Parkinson’s disease: Genetics and pathogenesis. Annu. Rev. Pathol..

[4] Hunot S., Hirsch E.C. (2003). Neuroinflammatory processes in Parkinson’s disease. Ann. Neurol..

[5] Kalinderi K., Bostantjopoulou S., Fidani L. (2016). The genetic background of Parkinson’s disease: Current progress and future prospects. Acta Neurol. Scand..

[6] Tambasco N., Nigro P., Romoli M., Prontera P., Simoni S., Calabresi P. (2016). A53T in a parkinsonian family: A clinical update of the SNCA phenotypes. J. Neural Transm..

[7] Deng H., Wang P., Jankovic J. (2018). The genetics of Parkinson disease. Ageing Res. Rev..

[8] de Lau L.M., Breteler M.M. (2006). Epidemiology of Parkinson’s disease. Lancet Neurol..

[9] Bergsland N., Zivadinov R., Schweser F., Hagemeier J., Lichter D., Guttuso T. (2019). Ventral posterior substantia nigra iron increases over 3 years in Parkinson’s disease. Mov. Disord..

[10] An H., Zeng X., Niu T., Li G., Yang J., Zheng L., Zhou W., Liu H., Zhang M., Huang D. (2018). Quantifying iron deposition within the substantia nigra of Parkinson’s disease by quantitative susceptibility mapping. J. Neurol. Sci..

[11] Tsalenchuk M., Gentleman S.M., Marzi S.J. (2023). Linking environmental risk factors with epigenetic mechanisms in Parkinson’s disease. NPJ Park. Dis..

[12] Costa H.N., Esteves A.R., Empadinhas N., Cardoso S.M. (2023). Parkinson’s Disease: A Multisystem Disorder. Neurosci. Bull..

[13] Hughes K.C., Gao X., Kim I.Y., Wang M., Weisskopf M.G., Schwarzschild M.A., Ascherio A. (2017). Intake of dairy foods and risk of Parkinson disease. Neurology.

[14] Domenighetti C., Sugier P.E., Ashok Kumar Sreelatha A., Schulte C., Grover S., Mohamed O., Portugal B., May P., Bobbili D.R., Radivojkov-Blagojevic M. (2022). Dairy Intake and Parkinson’s Disease: A Mendelian Randomization Study. Mov. Disord..

[15] Gronich N., Abernethy D.R., Auriel E., Lavi I., Rennert G., Saliba W. (2018). Beta2-adrenoceptor agonists and antagonists and risk of Parkinson’s disease. Mov. Disord..

[16] Searles Nielsen S., Gross A., Camacho-Soto A., Willis A.W., Racette B.A. (2018). Beta2-adrenoreceptor medications and risk of Parkinson disease. Ann. Neurol..

[17] Ross G.W., Abbott R.D., Petrovitch H., Morens D.M., Grandinetti A., Tung K.H., Tanner C.M., Masaki K.H., Blanchette P.L., Curb J.D. (2000). Association of coffee and caffeine intake with the risk of Parkinson disease. JAMA.

[18] Angelopoulou E., Stanitsa E., Karpodini C.C., Bougea A., Kontaxopoulou D., Fragkiadaki S., Koros C., Georgakopoulou V.E., Fotakopoulos G., Koutedakis Y. (2023). Pharmacological and Non-Pharmacological Treatments for Depression in Parkinson’s Disease: An Updated Review. Medicina.

[19] Smaili S., Hirata H., Ureshino R., Monteforte P.T., Morales A.P., Muler M.L., Terashima J., Oseki K., Rosenstock T.R., Lopes G.S. (2009). Calcium and cell death signaling in neurodegeneration and aging. Acad. Bras. Cienc..

[20] Lemasters J.J., Qian T., He L., Kim J.S., Elmore S.P., Cascio W.E., Brenner D.A. (2002). Role of mitochondrial inner membrane permeabilization in necrotic cell death, apoptosis, and autophagy. Antioxid. Redox Signal.

[21] Cali T., Ottolini D., Brini M. (2011). Mitochondria, calcium, and endoplasmic reticulum stress in Parkinson’s disease. Biofactors.

[22] Hirsch E.C., Vyas S., Hunot S. (2012). Neuroinflammation in Parkinson’s disease. Park. Relat. Disord..

[23] Surmeier D.J., Guzman J.N., Sanchez-Padilla J., Schumacker P.T. (2011). The role of calcium and mitochondrial oxidant stress in the loss of substantia nigra pars compacta dopaminergic neurons in Parkinson’s disease. Neuroscience.

[24] Trombetta-Lima M., Sabogal-Guaqueta A.M., Dolga A.M. (2021). Mitochondrial dysfunction in neurodegenerative diseases: A focus on iPSC-derived neuronal models. Cell Calcium.

[25] Teyler T.J. (1999). Use of brain slices to study long-term potentiation and depression as examples of synaptic plasticity. Methods.

[26] Schrader M., Godinho L.F., Costello J.L., Islinger M. (2015). The different facets of organelle interplay-an overview of organelle interactions. Front. Cell Dev. Biol..

[27] Han Y., Li M., Qiu F., Zhang M., Zhang Y.H. (2017). Cell-permeable organic fluorescent probes for live-cell long-term super-resolution imaging reveal lysosome-mitochondrion interactions. Nat. Commun..

[28] Tedeschi V., Petrozziello T., Secondo A. (2019). Calcium Dyshomeostasis and Lysosomal Ca^2+^ Dysfunction in Amyotrophic Lateral Sclerosis. Cells.

[29] Cali T., Ottolini D., Brini M. (2014). Calcium signaling in Parkinson’s disease. Cell Tissue Res..

[30] Patel S., Docampo R. (2010). Acidic calcium stores open for business: Expanding the potential for intracellular Ca^2+^ signaling. Trends Cell Biol..

[31] Bose A., Beal M.F. (2016). Mitochondrial dysfunction in Parkinson’s disease. J. Neurochem..

[32] Verma M., Callio J., Otero P.A., Sekler I., Wills Z.P., Chu C.T. (2017). Mitochondrial Calcium Dysregulation Contributes to Dendrite Degeneration Mediated by PD/LBD-Associated LRRK2 Mutants. J. Neurosci..

[33] Nagley P., Higgins G.C., Atkin J.D., Beart P.M. (2010). Multifaceted deaths orchestrated by mitochondria in neurones. Biochim. Biophys. Acta.

[34] Rcom-H’cheo-Gauthier A., Goodwin J., Pountney D.L. (2014). Interactions between calcium and alpha-synuclein in neurodegeneration. Biomolecules.

[35] Nath S., Goodwin J., Engelborghs Y., Pountney D.L. (2011). Raised calcium promotes alpha-synuclein aggregate formation. Mol. Cell. Neurosci..

[36] Caraveo G., Auluck P.K., Whitesell L., Chung C.Y., Baru V., Mosharov E.V., Yan X., Ben-Johny M., Soste M., Picotti P. (2014). Calcineurin determines toxic versus beneficial responses to alpha-synuclein. Proc. Natl. Acad. Sci. USA.

[37] Gomez-Sintes R., Ledesma M.D., Boya P. (2016). Lysosomal cell death mechanisms in aging. Ageing Res. Rev..

[38] Surmeier D.J., Obeso J.A., Halliday G.M. (2017). Selective neuronal vulnerability in Parkinson disease. Nat. Rev. Neurosci..

[39] Connors B.W., Gutnick M.J. (1990). Intrinsic firing patterns of diverse neocortical neurons. Trends Neurosci..

[40] Erisir A., Lau D., Rudy B., Leonard C.S. (1999). Function of specific K^+^ channels in sustained high-frequency firing of fast-spiking neocortical interneurons. J. Neurophysiol..

[41] Forti L., Cesana E., Mapelli J., D’Angelo E. (2006). Ionic mechanisms of autorhythmic firing in rat cerebellar Golgi cells. J. Physiol..

[42] Tateno T., Harsch A., Robinson H.P. (2004). Threshold firing frequency-current relationships of neurons in rat somatosensory cortex: Type 1 and type 2 dynamics. J. Neurophysiol..

[43] Bean B.P. (2007). The action potential in mammalian central neurons. Nat. Rev. Neurosci..

[44] Verkhratsky A., Nedergaard M. (2018). Physiology of Astroglia. Physiol. Rev..

[45] Schapira A.H. (2013). Calcium dysregulation in Parkinson’s disease. Brain.

[46] Surmeier D.J., Schumacker P.T., Guzman J.D., Ilijic E., Yang B., Zampese E. (2017). Calcium and Parkinson’s disease. Biochem. Biophys. Res. Commun..

[47] Zampese E., Surmeier D.J. (2020). Calcium, Bioenergetics, and Parkinson’s Disease. Cells.

[48] Di Martino R., Sisalli M.J., Sirabella R., Della Notte S., Borzacchiello D., Feliciello A., Annunziato L., Scorziello A. (2021). Ncx3-Induced Mitochondrial Dysfunction in Midbrain Leads to Neuroinflammation in Striatum of A53t-alpha-Synuclein Transgenic Old Mice. Int. J. Mol. Sci..

[49] Scorziello A., Borzacchiello D., Sisalli M.J., Di Martino R., Morelli M., Feliciello A. (2020). Mitochondrial Homeostasis and Signaling in Parkinson’s Disease. Front. Aging Neurosci..

[50] Visch H.J., Rutter G.A., Koopman W.J., Koenderink J.B., Verkaart S., de Groot T., Varadi A., Mitchell K.J., van den Heuvel L.P., Smeitink J.A. (2004). Inhibition of mitochondrial Na^+^-Ca^2+^ exchange restores agonist-induced ATP production and Ca^2+^ handling in human complex I deficiency. J. Biol. Chem..

[51] Philipson K.D., Nicoll D.A. (2000). Sodium-calcium exchange: A molecular perspective. Annu. Rev. Physiol..

[52] Lytton J. (2007). Na^+^/Ca^2+^ exchangers: Three mammalian gene families control Ca^2+^ transport. Biochem. J..

[53] On C., Marshall C.R., Chen N., Moyes C.D., Tibbits G.F. (2008). Gene structure evolution of the Na^+^-Ca^2+^ exchanger (NCX) family. BMC Evol. Biol..

[54] Nicoll D.A., Longoni S., Philipson K.D. (1990). Molecular cloning and functional expression of the cardiac sarcolemmal Na^+^-Ca^2+^ exchanger. Science.

[55] Lee S.L., Yu A.S., Lytton J. (1994). Tissue-specific expression of Na^+^-Ca^2+^ exchanger isoforms. J. Biol. Chem..

[56] Nicoll D.A., Quednau B.D., Qui Z., Xia Y.R., Lusis A.J., Philipson K.D. (1996). Cloning of a third mammalian Na^+^-Ca^2+^ exchanger, NCX3. J. Biol. Chem..

[57] Quednau B.D., Nicoll D.A., Philipson K.D. (1997). Tissue specificity and alternative splicing of the Na^+^/Ca^2+^ exchanger isoforms NCX1, NCX2, and NCX3 in rat. Am. J. Physiol..

[58] Khananshvili D., Weil-Maslansky E. (1994). The cardiac Na^+^-Ca^2+^ exchanger: Relative rates of calcium and sodium movements and their modulation by protonation-deprotonation of the carrier. Biochemistry.

[59] Khananshvili D. (2013). The SLC8 gene family of sodium-calcium exchangers (NCX)—Structure, function, and regulation in health and disease. Mol. Asp. Med..

[60] Blaustein M.P., Lederer W.J. (1999). Sodium/calcium exchange: Its physiological implications. Physiol. Rev..

[61] Kofuji P., Hadley R.W., Kieval R.S., Lederer W.J., Schulze D.H. (1992). Expression of the Na-Ca exchanger in diverse tissues: A study using the cloned human cardiac Na-Ca exchanger. Am. J. Physiol..

[62] Kofuji P., Lederer W.J., Schulze D.H. (1993). Na/Ca exchanger isoforms expressed in kidney. Am. J. Physiol..

[63] Matsuda T., Takuma K., Baba A. (1997). Na^+^-Ca^2+^ exchanger: Physiology and pharmacology. Jpn. J. Pharmacol..

[64] Li Z., Matsuoka S., Hryshko L.V., Nicoll D.A., Bersohn M.M., Burke E.P., Lifton R.P., Philipson K.D. (1994). Cloning of the NCX2 isoform of the plasma membrane Na^+^-Ca^2+^ exchanger. J. Biol. Chem..

[65] Papa M., Canitano A., Boscia F., Castaldo P., Sellitti S., Porzig H., Taglialatela M., Annunziato L. (2003). Differential expression of the Na^+^-Ca^2+^ exchanger transcripts and proteins in rat brain regions. J. Comp. Neurol..

[66] Reeves J.P., Hale C.C. (1984). The stoichiometry of the cardiac sodium-calcium exchange system. J. Biol. Chem..

[67] Piccirillo S., Castaldo P., Macri M.L., Amoroso S., Magi S. (2018). Glutamate as a potential “survival factor” in an in vitro model of neuronal hypoxia/reoxygenation injury: Leading role of the Na^+^/Ca^2+^ exchanger. Cell Death Dis..

[68] Wood-Kaczmar A., Deas E., Wood N.W., Abramov A.Y. (2013). The role of the mitochondrial NCX in the mechanism of neurodegeneration in Parkinson’s disease. Adv. Exp. Med. Biol..

[69] Annunziato L., Pignataro G., Di Renzo G.F. (2004). Pharmacology of brain Na^+^/Ca^2+^ exchanger: From molecular biology to therapeutic perspectives. Pharmacol. Rev..

[70] Maiolino M., Castaldo P., Lariccia V., Piccirillo S., Amoroso S., Magi S. (2017). Essential role of the Na^+^-Ca^2+^ exchanger (NCX) in glutamate-enhanced cell survival in cardiac cells exposed to hypoxia/reoxygenation. Sci. Rep..

[71] Bastioli G., Piccirillo S., Castaldo P., Magi S., Tozzi A., Amoroso S., Calabresi P. (2019). Selective inhibition of mitochondrial sodium-calcium exchanger protects striatal neurons from alpha-synuclein plus rotenone induced toxicity. Cell Death Dis..

[72] Piccirillo S., Magi S., Preziuso A., Castaldo P., Amoroso S., Lariccia V. (2020). Gateways for Glutamate Neuroprotection in Parkinson’s Disease (PD): Essential Role of EAAT3 and NCX1 Revealed in an In Vitro Model of PD. Cells.

[73] Magi S., Piccirillo S., Preziuso A., Amoroso S., Lariccia V. (2020). Mitochondrial localization of NCXs: Balancing calcium and energy homeostasis. Cell Calcium.

[74] Magi S., Preziuso A., Piccirillo S., Giampieri F., Cianciosi D., Orciani M., Amoroso S. (2021). The Neuroprotective Effect of L-Carnitine against Glyceraldehyde-Induced Metabolic Impairment: Possible Implications in Alzheimer’s Disease. Cells.

[75] Preziuso A., Piccirillo S., Cerqueni G., Serfilippi T., Terenzi V., Vinciguerra A., Orciani M., Amoroso S., Magi S., Lariccia V. (2023). Exploring the Role of NCX1 and NCX3 in an In Vitro Model of Metabolism Impairment: Potential Neuroprotective Targets for Alzheimer’s Disease. Biology.

[76] Pignataro G., Gala R., Cuomo O., Tortiglione A., Giaccio L., Castaldo P., Sirabella R., Matrone C., Canitano A., Amoroso S. (2004). Two sodium/calcium exchanger gene products, NCX1 and NCX3, play a major role in the development of permanent focal cerebral ischemia. Stroke.

[77] Matsuda T., Arakawa N., Takuma K., Kishida Y., Kawasaki Y., Sakaue M., Takahashi K., Takahashi T., Suzuki T., Ota T. (2001). SEA0400, a novel and selective inhibitor of the Na^+^-Ca^2+^ exchanger, attenuates reperfusion injury in the in vitro and in vivo cerebral ischemic models. J. Pharmacol. Exp. Ther..

[78] Canitano A., Papa M., Boscia F., Castaldo P., Sellitti S., Taglialatela M., Annunziato L. (2002). Brain distribution of the Na^+^/Ca^2+^ exchanger-encoding genes NCX1, NCX2, and NCX3 and their related proteins in the central nervous system. Ann. N. Y. Acad. Sci..

[79] Jackson-Lewis V., Przedborski S. (2007). Protocol for the MPTP mouse model of Parkinson’s disease. Nat. Protoc..

[80] Ago Y., Kawasaki T., Nashida T., Ota Y., Cong Y., Kitamoto M., Takahashi T., Takuma K., Matsuda T. (2011). SEA0400, a specific Na^+^/Ca^2+^ exchange inhibitor, prevents dopaminergic neurotoxicity in an MPTP mouse model of Parkinson’s disease. Neuropharmacology.

[81] Sirabella R., Sisalli M.J., Costa G., Omura K., Ianniello G., Pinna A., Morelli M., Di Renzo G.M., Annunziato L., Scorziello A. (2018). NCX1 and NCX3 as potential factors contributing to neurodegeneration and neuroinflammation in the A53T transgenic mouse model of Parkinson’s Disease. Cell Death Dis..

[82] Friedman J.R., Nunnari J. (2014). Mitochondrial form and function. Nature.

[83] Nicholls D. (2002). Mitochondrial bioenergetics, aging, and aging-related disease. Sci. Aging Knowl. Environ..

[84] Brandon M., Baldi P., Wallace D.C. (2006). Mitochondrial mutations in cancer. Oncogene.

[85] Szydlowska K., Tymianski M. (2010). Calcium, ischemia and excitotoxicity. Cell Calcium.

[86] Jin H., Kanthasamy A., Ghosh A., Anantharam V., Kalyanaraman B., Kanthasamy A.G. (2014). Mitochondria-targeted antioxidants for treatment of Parkinson’s disease: Preclinical and clinical outcomes. Biochim. Biophys. Acta.

[87] Banerjee P., Saha I., Sarkar D., Maiti A.K. (2022). Contributions and Limitations of Mitochondria-Targeted and Non-Targeted Antioxidants in the Treatment of Parkinsonism: An Updated Review. Neurotox. Res..

[88] Subramaniam S.R., Chesselet M.F. (2013). Mitochondrial dysfunction and oxidative stress in Parkinson’s disease. Prog. Neurobiol..

[89] Ludtmann M.H.R., Angelova P.R., Horrocks M.H., Choi M.L., Rodrigues M., Baev A.Y., Berezhnov A.V., Yao Z., Little D., Banushi B. (2018). alpha-synuclein oligomers interact with ATP synthase and open the permeability transition pore in Parkinson’s disease. Nat. Commun..

[90] Chung C.Y., Khurana V., Auluck P.K., Tardiff D.F., Mazzulli J.R., Soldner F., Baru V., Lou Y., Freyzon Y., Cho S. (2013). Identification and rescue of alpha-synuclein toxicity in Parkinson patient-derived neurons. Science.

[91] Zambon F., Cherubini M., Fernandes H.J.R., Lang C., Ryan B.J., Volpato V., Bengoa-Vergniory N., Vingill S., Attar M., Booth H.D.E. (2019). Cellular alpha-synuclein pathology is associated with bioenergetic dysfunction in Parkinson’s iPSC-derived dopamine neurons. Hum. Mol. Genet..

[92] Mahul-Mellier A.L., Burtscher J., Maharjan N., Weerens L., Croisier M., Kuttler F., Leleu M., Knott G.W., Lashuel H.A. (2020). The process of Lewy body formation, rather than simply alpha-synuclein fibrillization, is one of the major drivers of neurodegeneration. Proc. Natl. Acad. Sci. USA.

[93] Luth E.S., Stavrovskaya I.G., Bartels T., Kristal B.S., Selkoe D.J. (2014). Soluble, prefibrillar alpha-synuclein oligomers promote complex I-dependent, Ca^2+^-induced mitochondrial dysfunction. J. Biol. Chem..

[94] Sliter D.A., Martinez J., Hao L., Chen X., Sun N., Fischer T.D., Burman J.L., Li Y., Zhang Z., Narendra D.P. (2018). Parkin and PINK1 mitigate STING-induced inflammation. Nature.

[95] Beccano-Kelly D.A., Cherubini M., Mousba Y., Cramb K.M.L., Giussani S., Caiazza M.C., Rai P., Vingill S., Bengoa-Vergniory N., Ng B. (2023). Calcium dysregulation combined with mitochondrial failure and electrophysiological maturity converge in Parkinson’s iPSC-dopamine neurons. iScience.

[96] Castaldo P., Cataldi M., Magi S., Lariccia V., Arcangeli S., Amoroso S. (2009). Role of the mitochondrial sodium/calcium exchanger in neuronal physiology and in the pathogenesis of neurological diseases. Prog. Neurobiol..

[97] Carafoli E., Tiozzo R., Lugli G., Crovetti F., Kratzing C. (1974). The release of calcium from heart mitochondria by sodium. J. Mol. Cell Cardiol..

[98] Crompton M., Kunzi M., Carafoli E. (1977). The calcium-induced and sodium-induced effluxes of calcium from heart mitochondria. Evidence for a sodium-calcium carrier. Eur. J. Biochem..

[99] Palty R., Ohana E., Hershfinkel M., Volokita M., Elgazar V., Beharier O., Silverman W.F., Argaman M., Sekler I. (2004). Lithium-calcium exchange is mediated by a distinct potassium-independent sodium-calcium exchanger. J. Biol. Chem..

[100] Palty R., Silverman W.F., Hershfinkel M., Caporale T., Sensi S.L., Parnis J., Nolte C., Fishman D., Shoshan-Barmatz V., Herrmann S. (2010). NCLX is an essential component of mitochondrial Na^+^/Ca^2+^ exchange. Proc. Natl. Acad. Sci. USA.

[101] Gobbi P., Castaldo P., Minelli A., Salucci S., Magi S., Corcione E., Amoroso S. (2007). Mitochondrial localization of Na^+^/Ca^2+^ exchangers NCX1-3 in neurons and astrocytes of adult rat brain in situ. Pharmacol. Res..

[102] Minelli A., Castaldo P., Gobbi P., Salucci S., Magi S., Amoroso S. (2007). Cellular and subcellular localization of Na^+^-Ca^2+^ exchanger protein isoforms, NCX1, NCX2, and NCX3 in cerebral cortex and hippocampus of adult rat. Cell Calcium.

[103] Magi S., Lariccia V., Castaldo P., Arcangeli S., Nasti A.A., Giordano A., Amoroso S. (2012). Physical and functional interaction of NCX1 and EAAC1 transporters leading to glutamate-enhanced ATP production in brain mitochondria. PLoS ONE.

[104] Scorziello A., Savoia C., Sisalli M.J., Adornetto A., Secondo A., Boscia F., Esposito A., Polishchuk E.V., Polishchuk R.S., Molinaro P. (2013). NCX3 regulates mitochondrial Ca^2+^ handling through the AKAP121-anchored signaling complex and prevents hypoxia-induced neuronal death. J. Cell Sci..

[105] Sharma V., He C., Sacca-Schaeffer J., Brzozowski E., Martin-Herranz D.E., Mendelowitz Z., Fitzpatrick D.A., O’Halloran D.M. (2013). Insight into the family of Na^+^/Ca^2+^ exchangers of Caenorhabditis elegans. Genetics.

[106] Sharma V., O’Halloran D.M. (2016). Nematode Sodium Calcium Exchangers: A Surprising Lack of Transport. Bioinform. Biol. Insights.

[107] Gandhi S., Wood-Kaczmar A., Yao Z., Plun-Favreau H., Deas E., Klupsch K., Downward J., Latchman D.S., Tabrizi S.J., Wood N.W. (2009). PINK1-associated Parkinson’s disease is caused by neuronal vulnerability to calcium-induced cell death. Mol. Cell.

[108] Heeman B., Van den Haute C., Aelvoet S.A., Valsecchi F., Rodenburg R.J., Reumers V., Debyser Z., Callewaert G., Koopman W.J., Willems P.H. (2011). Depletion of PINK1 affects mitochondrial metabolism, calcium homeostasis and energy maintenance. J. Cell Sci..

[109] Kostic M., Ludtmann M.H., Bading H., Hershfinkel M., Steer E., Chu C.T., Abramov A.Y., Sekler I. (2015). PKA Phosphorylation of NCLX Reverses Mitochondrial Calcium Overload and Depolarization, Promoting Survival of PINK1-Deficient Dopaminergic Neurons. Cell Rep..

[110] Ludtmann M.H.R., Kostic M., Horne A., Gandhi S., Sekler I., Abramov A.Y. (2019). LRRK2 deficiency induced mitochondrial Ca^2+^ efflux inhibition can be rescued by Na^+^/Ca^2+^/Li^+^ exchanger upregulation. Cell Death Dis..

[111] Wang M., Kaufman R.J. (2016). Protein misfolding in the endoplasmic reticulum as a conduit to human disease. Nature.

[112] Ghemrawi R., Khair M. (2020). Endoplasmic Reticulum Stress and Unfolded Protein Response in Neurodegenerative Diseases. Int. J. Mol. Sci..

[113] Bahar E., Kim H., Yoon H. (2016). ER Stress-Mediated Signaling: Action Potential and Ca^2+^ as Key Players. Int. J. Mol. Sci..

[114] Bezprozvanny I.B. (2010). Calcium signaling and neurodegeneration. Acta Naturae.

[115] Bootman M.D. (2012). Calcium signaling. Cold Spring Harb. Perspect. Biol..

[116] Carafoli E. (2002). Calcium signaling: A tale for all seasons. Proc. Natl. Acad. Sci. USA.

[117] McBrayer M., Nixon R.A. (2013). Lysosome and calcium dysregulation in Alzheimer’s disease: Partners in crime. Biochem. Soc. Trans..

[118] Phillips M.J., Voeltz G.K. (2016). Structure and function of ER membrane contact sites with other organelles. Nat. Rev. Mol. Cell Biol..

[119] Rivero-Rios P., Gomez-Suaga P., Fdez E., Hilfiker S. (2014). Upstream deregulation of calcium signaling in Parkinson’s disease. Front. Mol. Neurosci..

[120] Wojda U., Salinska E., Kuznicki J. (2008). Calcium ions in neuronal degeneration. IUBMB Life.

[121] Zaichick S.V., McGrath K.M., Caraveo G. (2017). The role of Ca^2+^ signaling in Parkinson’s disease. Dis. Model. Mech..

[122] Prins D., Michalak M. (2011). Organellar calcium buffers. Cold Spring Harb. Perspect. Biol..

[123] Rodriguez-Arribas M., Yakhine-Diop S.M.S., Pedro J.M.B., Gomez-Suaga P., Gomez-Sanchez R., Martinez-Chacon G., Fuentes J.M., Gonzalez-Polo R.A., Niso-Santano M. (2017). Mitochondria-Associated Membranes (MAMs): Overview and Its Role in Parkinson’s Disease. Mol. Neurobiol..

[124] Grossmann D., Malburg N., Glass H., Weeren V., Sondermann V., Pfeiffer J.F., Petters J., Lukas J., Seibler P., Klein C. (2023). Mitochondria-Endoplasmic Reticulum Contact Sites Dynamics and Calcium Homeostasis Are Differentially Disrupted in PINK1-PD or PRKN-PD Neurons. Mov. Disord..

[125] Zhao Y., Shen W., Zhang M., Guo M., Dou Y., Han S., Yu J., Cui M., Zhao Y. (2024). DDAH-1 maintains endoplasmic reticulum-mitochondria contacts and protects dopaminergic neurons in Parkinson’s disease. Cell Death Dis..

[126] Gautier C.A., Erpapazoglou Z., Mouton-Liger F., Muriel M.P., Cormier F., Bigou S., Duffaure S., Girard M., Foret B., Iannielli A. (2016). The endoplasmic reticulum-mitochondria interface is perturbed in PARK2 knockout mice and patients with PARK2 mutations. Hum. Mol. Genet..

[127] Mercado G., Valdes P., Hetz C. (2013). An ERcentric view of Parkinson’s disease. Trends Mol. Med..

[128] Nixon R.A. (2013). The role of autophagy in neurodegenerative disease. Nat. Med..

[129] Gomez-Suaga P., Bravo-San Pedro J.M., Gonzalez-Polo R.A., Fuentes J.M., Niso-Santano M. (2018). ER-mitochondria signaling in Parkinson’s disease. Cell Death Dis..

[130] Carreras-Sureda A., Pihan P., Hetz C. (2018). Calcium signaling at the endoplasmic reticulum: Fine-tuning stress responses. Cell Calcium.

[131] Healy D.G., Abou-Sleiman P.M., Wood N.W. (2004). PINK, PANK, or PARK? A clinicians’ guide to familial parkinsonism. Lancet Neurol..

[132] Greenamyre J.T., Hastings T.G. (2004). Biomedicine. Parkinson’s—Divergent causes, convergent mechanisms. Science.

[133] Betarbet R., Sherer T.B., MacKenzie G., Garcia-Osuna M., Panov A.V., Greenamyre J.T. (2000). Chronic systemic pesticide exposure reproduces features of Parkinson’s disease. Nat. Neurosci..

[134] Shen J. (2004). Protein kinases linked to the pathogenesis of Parkinson’s disease. Neuron.

[135] Ugolino J., Fang S., Kubisch C., Monteiro M.J. (2011). Mutant Atp13a2 proteins involved in parkinsonism are degraded by ER-associated degradation and sensitize cells to ER-stress induced cell death. Hum. Mol. Genet..

[136] Tomiyama H., Yoshino H., Ogaki K., Li L., Yamashita C., Li Y., Funayama M., Sasaki R., Kokubo Y., Kuzuhara S. (2011). PLA2G6 variant in Parkinson’s disease. J. Hum. Genet..

[137] Schneider L., Zhang J. (2010). Lysosomal function in macromolecular homeostasis and bioenergetics in Parkinson’s disease. Mol. Neurodegener..

[138] Tedeschi V., Secondo A. (2022). Emerging role of lysosomal calcium store as a hub of neuroprotection. Neural Regen. Res..

[139] Patel S., Muallem S. (2011). Acidic Ca^2+^ stores come to the fore. Cell Calcium.

[140] Schondorf D.C., Aureli M., McAllister F.E., Hindley C.J., Mayer F., Schmid B., Sardi S.P., Valsecchi M., Hoffmann S., Schwarz L.K. (2014). iPSC-derived neurons from GBA1-associated Parkinson’s disease patients show autophagic defects and impaired calcium homeostasis. Nat. Commun..

[141] Gomez-Suaga P., Luzon-Toro B., Churamani D., Zhang L., Bloor-Young D., Patel S., Woodman P.G., Churchill G.C., Hilfiker S. (2012). Leucine-rich repeat kinase 2 regulates autophagy through a calcium-dependent pathway involving NAADP. Hum. Mol. Genet..

[142] Tsunemi T., Perez-Rosello T., Ishiguro Y., Yoroisaka A., Jeon S., Hamada K., Rammonhan M., Wong Y.C., Xie Z., Akamatsu W. (2019). Increased Lysosomal Exocytosis Induced by Lysosomal Ca^2+^ Channel Agonists Protects Human Dopaminergic Neurons from alpha-Synuclein Toxicity. J. Neurosci..

[143] Scotto Rosato A., Montefusco S., Soldati C., Di Paola S., Capuozzo A., Monfregola J., Polishchuk E., Amabile A., Grimm C., Lombardo A. (2019). TRPML1 links lysosomal calcium to autophagosome biogenesis through the activation of the CaMKKbeta/VPS34 pathway. Nat. Commun..

[144] Santoni G., Morelli M.B., Amantini C., Nabissi M., Santoni M., Santoni A. (2020). Involvement of the TRPML Mucolipin Channels in Viral Infections and Anti-viral Innate Immune Responses. Front. Immunol..

[145] Gitler A.D., Chesi A., Geddie M.L., Strathearn K.E., Hamamichi S., Hill K.J., Caldwell K.A., Caldwell G.A., Cooper A.A., Rochet J.C. (2009). Alpha-synuclein is part of a diverse and highly conserved interaction network that includes PARK9 and manganese toxicity. Nat. Genet..

[146] Tsunemi T., Hamada K., Krainc D. (2014). ATP13A2/PARK9 regulates secretion of exosomes and alpha-synuclein. J. Neurosci..

[147] Lopes da Fonseca T., Outeiro T.F. (2014). ATP13A2 and Alpha-synuclein: A Metal Taste in Autophagy. Exp. Neurobiol..

[148] Bento C.F., Ashkenazi A., Jimenez-Sanchez M., Rubinsztein D.C. (2016). The Parkinson’s disease-associated genes ATP13A2 and SYT11 regulate autophagy via a common pathway. Nat. Commun..

[149] Zhang X., Cheng X., Yu L., Yang J., Calvo R., Patnaik S., Hu X., Gao Q., Yang M., Lawas M. (2016). MCOLN1 is a ROS sensor in lysosomes that regulates autophagy. Nat. Commun..

[150] Zhang L., Fang Y., Zhao X., Zheng Y., Ma Y., Li S., Huang Z., Li L. (2021). miR-204 silencing reduces mitochondrial autophagy and ROS production in a murine AD model via the TRPML1-activated STAT3 pathway. Mol. Ther. Nucleic Acids.

[151] Sardiello M., Palmieri M., di Ronza A., Medina D.L., Valenza M., Gennarino V.A., Di Malta C., Donaudy F., Embrione V., Polishchuk R.S. (2009). A gene network regulating lysosomal biogenesis and function. Science.

[152] Medina D.L., Di Paola S., Peluso I., Armani A., De Stefani D., Venditti R., Montefusco S., Scotto-Rosato A., Prezioso C., Forrester A. (2015). Lysosomal calcium signalling regulates autophagy through calcineurin and TFEB. Nat. Cell Biol..

[153] Decressac M., Mattsson B., Weikop P., Lundblad M., Jakobsson J., Bjorklund A. (2013). TFEB-mediated autophagy rescues midbrain dopamine neurons from alpha-synuclein toxicity. Proc. Natl. Acad. Sci. USA.

[154] Torra A., Parent A., Cuadros T., Rodriguez-Galvan B., Ruiz-Bronchal E., Ballabio A., Bortolozzi A., Vila M., Bove J. (2018). Overexpression of TFEB Drives a Pleiotropic Neurotrophic Effect and Prevents Parkinson’s Disease-Related Neurodegeneration. Mol. Ther..

[155] Zhuang X.X., Wang S.F., Tan Y., Song J.X., Zhu Z., Wang Z.Y., Wu M.Y., Cai C.Z., Huang Z.J., Tan J.Q. (2020). Pharmacological enhancement of TFEB-mediated autophagy alleviated neuronal death in oxidative stress-induced Parkinson’s disease models. Cell Death Dis..

[156] Zhang L., Fang Y., Cheng X., Lian Y., Xu H., Zeng Z., Zhu H. (2017). TRPML1 Participates in the Progression of Alzheimer’s Disease by Regulating the PPARgamma/AMPK/Mtor Signalling Pathway. Cell Physiol. Biochem..

[157] Neudorfer O., Giladi N., Elstein D., Abrahamov A., Turezkite T., Aghai E., Reches A., Bembi B., Zimran A. (1996). Occurrence of Parkinson’s syndrome in type I Gaucher disease. QJM.

[158] Mazzulli J.R., Zunke F., Tsunemi T., Toker N.J., Jeon S., Burbulla L.F., Patnaik S., Sidransky E., Marugan J.J., Sue C.M. (2016). Activation of beta-Glucocerebrosidase Reduces Pathological alpha-Synuclein and Restores Lysosomal Function in Parkinson’s Patient Midbrain Neurons. J. Neurosci..

[159] Davalos D., Grutzendler J., Yang G., Kim J.V., Zuo Y., Jung S., Littman D.R., Dustin M.L., Gan W.B. (2005). ATP mediates rapid microglial response to local brain injury in vivo. Nat. Neurosci..

[160] Nimmerjahn A., Kirchhoff F., Helmchen F. (2005). Resting microglial cells are highly dynamic surveillants of brain parenchyma in vivo. Science.

[161] Wolf S.A., Boddeke H.W., Kettenmann H. (2017). Microglia in Physiology and Disease. Annu. Rev. Physiol..

[162] Szalay G., Martinecz B., Lenart N., Kornyei Z., Orsolits B., Judak L., Csaszar E., Fekete R., West B.L., Katona G. (2016). Microglia protect against brain injury and their selective elimination dysregulates neuronal network activity after stroke. Nat. Commun..

[163] Wake H., Moorhouse A.J., Jinno S., Kohsaka S., Nabekura J. (2009). Resting microglia directly monitor the functional state of synapses in vivo and determine the fate of ischemic terminals. J. Neurosci..

[164] Akiyoshi R., Wake H., Kato D., Horiuchi H., Ono R., Ikegami A., Haruwaka K., Omori T., Tachibana Y., Moorhouse A.J. (2018). Microglia Enhance Synapse Activity to Promote Local Network Synchronization. eNeuro.

[165] Miyamoto A., Wake H., Ishikawa A.W., Eto K., Shibata K., Murakoshi H., Koizumi S., Moorhouse A.J., Yoshimura Y., Nabekura J. (2016). Microglia contact induces synapse formation in developing somatosensory cortex. Nat. Commun..

[166] Reshef R., Kudryavitskaya E., Shani-Narkiss H., Isaacson B., Rimmerman N., Mizrahi A., Yirmiya R. (2017). The role of microglia and their CX3CR1 signaling in adult neurogenesis in the olfactory bulb. eLife.

[167] Hinks G.L., Franklin R.J. (1999). Distinctive patterns of PDGF-A, FGF-2, IGF-I, and TGF-beta1 gene expression during remyelination of experimentally-induced spinal cord demyelination. Mol. Cell Neurosci..

[168] Verkhratsky A., Zorec R., Rodriguez J.J., Parpura V. (2017). Neuroglia: Functional Paralysis and Reactivity in Alzheimer’s Disease and Other Neurodegenerative Pathologies. Adv. Neurobiol..

[169] Muzio L., Viotti A., Martino G. (2021). Microglia in Neuroinflammation and Neurodegeneration: From Understanding to Therapy. Front. Neurosci..

[170] Tansey M.G., Wallings R.L., Houser M.C., Herrick M.K., Keating C.E., Joers V. (2022). Inflammation and immune dysfunction in Parkinson disease. Nat. Rev. Immunol..

[171] Bsibsi M., Ravid R., Gveric D., van Noort J.M. (2002). Broad expression of Toll-like receptors in the human central nervous system. J. Neuropathol. Exp. Neurol..

[172] Olson J.K., Miller S.D. (2004). Microglia initiate central nervous system innate and adaptive immune responses through multiple TLRs. J. Immunol..

[173] Piccinini A.M., Midwood K.S. (2010). DAMPening inflammation by modulating TLR signalling. Mediat. Inflamm..

[174] Matzinger P., Kamala T. (2011). Tissue-based class control: The other side of tolerance. Nat. Rev. Immunol..

[175] Rojo A.I., McBean G., Cindric M., Egea J., Lopez M.G., Rada P., Zarkovic N., Cuadrado A. (2014). Redox control of microglial function: Molecular mechanisms and functional significance. Antioxid. Redox Signal..

[176] Yang Y., Song J.J., Choi Y.R., Kim S.H., Seok M.J., Wulansari N., Darsono W.H.W., Kwon O.C., Chang M.Y., Park S.M. (2022). Therapeutic functions of astrocytes to treat alpha-synuclein pathology in Parkinson’s disease. Proc. Natl. Acad. Sci. USA.

[177] Prunell G., Olivera-Bravo S. (2022). A Focus on Astrocyte Contribution to Parkinson’s Disease Etiology. Biomolecules.

[178] Butler C.A., Popescu A.S., Kitchener E.J.A., Allendorf D.H., Puigdellivol M., Brown G.C. (2021). Microglial phagocytosis of neurons in neurodegeneration, and its regulation. J. Neurochem..

[179] Zhang W., Wang T., Pei Z., Miller D.S., Wu X., Block M.L., Wilson B., Zhang W., Zhou Y., Hong J.S. (2005). Aggregated alpha-synuclein activates microglia: A process leading to disease progression in Parkinson’s disease. FASEB J..

[180] Park J.Y., Paik S.R., Jou I., Park S.M. (2008). Microglial phagocytosis is enhanced by monomeric alpha-synuclein, not aggregated alpha-synuclein: Implications for Parkinson’s disease. Glia.

[181] Rogers J., Mastroeni D., Leonard B., Joyce J., Grover A. (2007). Neuroinflammation in Alzheimer’s disease and Parkinson’s disease: Are microglia pathogenic in either disorder?. Int. Rev. Neurobiol..

[182] Booth H.D.E., Hirst W.D., Wade-Martins R. (2017). The Role of Astrocyte Dysfunction in Parkinson’s Disease Pathogenesis. Trends Neurosci..

[183] Bancroft E.A., Srinivasan R. (2021). Emerging Roles for Aberrant Astrocytic Calcium Signals in Parkinson’s Disease. Front. Physiol..

[184] Sonninen T.M., Hamalainen R.H., Koskuvi M., Oksanen M., Shakirzyanova A., Wojciechowski S., Puttonen K., Naumenko N., Goldsteins G., Laham-Karam N. (2020). Metabolic alterations in Parkinson’s disease astrocytes. Sci. Rep..

[185] Barkholt P., Sanchez-Guajardo V., Kirik D., Romero-Ramos M. (2012). Long-term polarization of microglia upon alpha-synuclein overexpression in nonhuman primates. Neuroscience.

[186] Liddelow S.A., Guttenplan K.A., Clarke L.E., Bennett F.C., Bohlen C.J., Schirmer L., Bennett M.L., Munch A.E., Chung W.S., Peterson T.C. (2017). Neurotoxic reactive astrocytes are induced by activated microglia. Nature.

[187] Lee H.J., Kim C., Lee S.J. (2010). Alpha-synuclein stimulation of astrocytes: Potential role for neuroinflammation and neuroprotection. Oxidative Med. Cell Longev..

[188] Thi Lai T., Kim Y.E., Nguyen L.T.N., Thi Nguyen T., Kwak I.H., Richter F., Kim Y.J., Ma H.I. (2024). Microglial inhibition alleviates alpha-synuclein propagation and neurodegeneration in Parkinson’s disease mouse model. NPJ Park. Dis..

[189] Lev N., Barhum Y., Ben-Zur T., Melamed E., Steiner I., Offen D. (2013). Knocking out DJ-1 attenuates astrocytes neuroprotection against 6-hydroxydopamine toxicity. J. Mol. Neurosci..

[190] Park J., Choi H., Min J.S., Park S.J., Kim J.H., Park H.J., Kim B., Chae J.I., Yim M., Lee D.S. (2013). Mitochondrial dynamics modulate the expression of pro-inflammatory mediators in microglial cells. J. Neurochem..

[191] Bordt E.A., Polster B.M. (2014). NADPH oxidase- and mitochondria-derived reactive oxygen species in proinflammatory microglial activation: A bipartisan affair?. Free Radic. Biol. Med..

[192] Ramos-Gonzalez P., Mato S., Chara J.C., Verkhratsky A., Matute C., Cavaliere F. (2021). Astrocytic atrophy as a pathological feature of Parkinson’s disease with LRRK2 mutation. NPJ Park. Dis..

[193] Bastioli G., Regoni M., Cazzaniga F., De Luca C.M.G., Bistaffa E., Zanetti L., Moda F., Valtorta F., Sassone J. (2021). Animal Models of Autosomal Recessive Parkinsonism. Biomedicines.

[194] Zhang Y., Meng X., Jiao Z., Liu Y., Zhang X., Qu S. (2020). Generation of a Novel Mouse Model of Parkinson’s Disease via Targeted Knockdown of Glutamate Transporter GLT-1 in the Substantia Nigra. ACS Chem. Neurosci..

[195] Chotibut T., Meadows S., Kasanga E.A., McInnis T., Cantu M.A., Bishop C., Salvatore M.F. (2017). Ceftriaxone reduces L-dopa-induced dyskinesia severity in 6-hydroxydopamine Parkinson’s disease model. Mov. Disord..

[196] Ibanez I., Bartolome-Martin D., Piniella D., Gimenez C., Zafra F. (2019). Activity dependent internalization of the glutamate transporter GLT-1 requires calcium entry through the NCX sodium/calcium exchanger. Neurochem. Int..

[197] Adermark L., Lagstrom O., Loften A., Licheri V., Havenang A., Loi E.A., Stomberg R., Soderpalm B., Domi A., Ericson M. (2022). Astrocytes modulate extracellular neurotransmitter levels and excitatory neurotransmission in dorsolateral striatum via dopamine D2 receptor signaling. Neuropsychopharmacology.

[198] Corkrum M., Covelo A., Lines J., Bellocchio L., Pisansky M., Loke K., Quintana R., Rothwell P.E., Lujan R., Marsicano G. (2020). Dopamine-Evoked Synaptic Regulation in the Nucleus Accumbens Requires Astrocyte Activity. Neuron.

[199] Xin W., Schuebel K.E., Jair K.W., Cimbro R., De Biase L.M., Goldman D., Bonci A. (2019). Ventral midbrain astrocytes display unique physiological features and sensitivity to dopamine D2 receptor signaling. Neuropsychopharmacology.

